# Supplementary material for: Genome-Wide Identification and Characterization of MYB Gene Family and Analysis of Its Sex-Biased Expression Pattern in Spinacia oleracea L
Source: Int J Mol Sci. 2024 Jan 8;25(2):795. doi: 10.3390/ijms25020795 (PMC10815031; doi:10.3390/ijms25020795)
Supplement: Supplementary file 1 [file ijms-25-00795-s001.zip › Supplementary data.pdf]

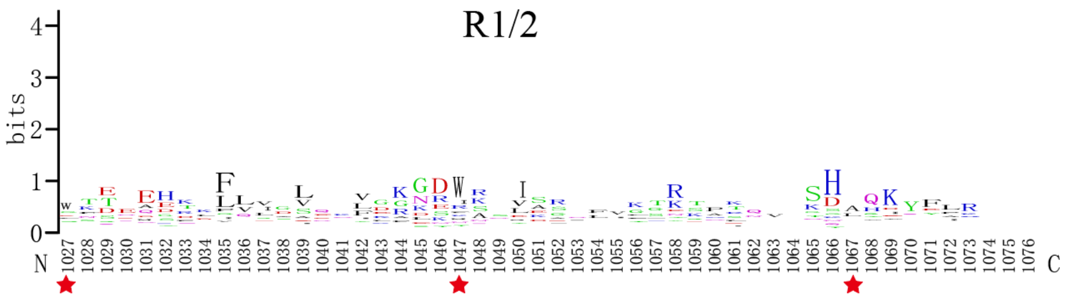

Figure S1. Domain sequence characteristics of 1R-MYB in spinach. The red stars represent the position of tryptophan. The WebLogo online website was used to visualize the Sequence logo.

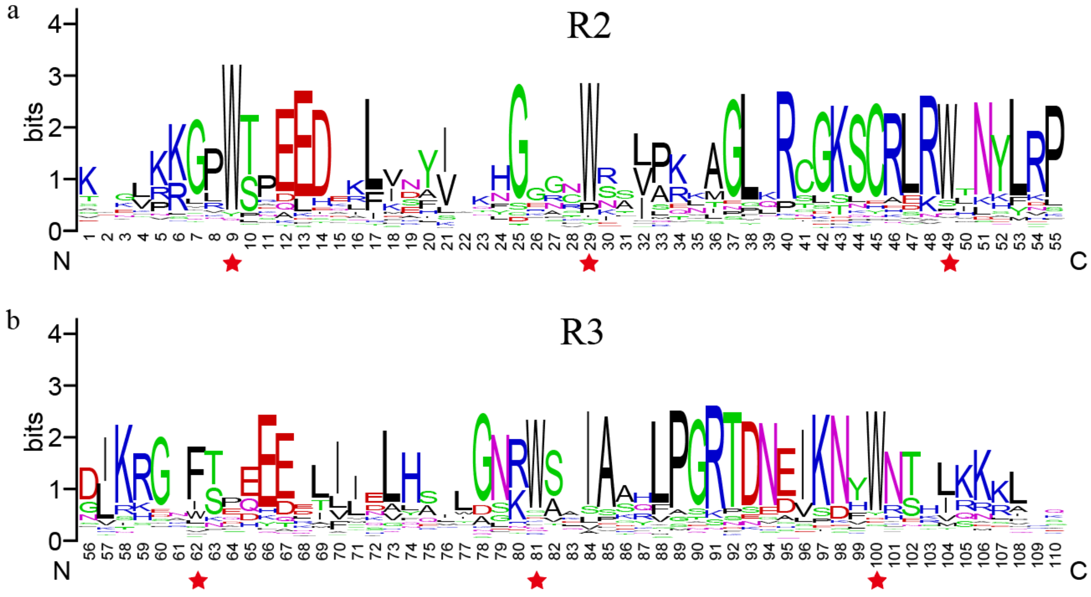

Figure S2. Domain sequence characteristics of 2R-MYB (a: R2 domain; b: R3 domain). The red stars represent the position of tryptophan. The WebLogo online website was used to visualize the Sequence logo.

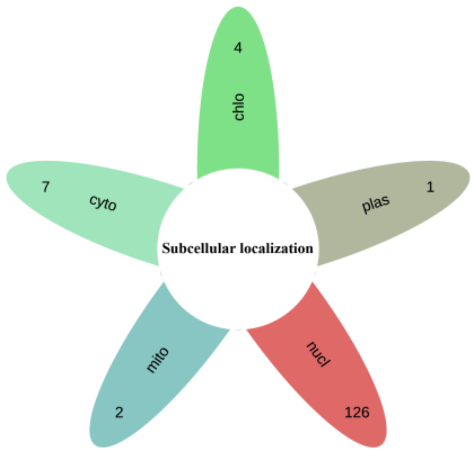

Figure S3. Prediction results of MYB gene subcellular localization in spinach. Different color petals represent different localization, and the number represents the number of genes predicted to be localized. The online site WoLF PSORT was used for subcellular localization prediction and

the EVeen online site was used for visualization.
